# Implementation of a study to examine the persistence of Ebola virus in the body fluids of Ebola virus disease survivors in Sierra Leone: Methodology and lessons learned

**DOI:** 10.1371/journal.pntd.0005723

**Published:** 2017-09-11

**Authors:** Gibrilla Fadlu Deen, Suzanna L. R. McDonald, Jaclyn E. Marrinan, Foday R. Sesay, Elizabeth Ervin, Anna E. Thorson, Wenbo Xu, Ute Ströher, Patricia Ongpin, Neetu Abad, Archchun Ariyarajah, Tasneem Malik, Hongtu Liu, Christine Ross, Kara N. Durski, Philippe Gaillard, Oliver Morgan, Pierre Formenty, Barbara Knust, Nathalie Broutet, Foday Sahr, on behalf of the Sierra Leone Ebola Virus Persistence Study Group

**Affiliations:** 1 Clinical Studies, Internal Medicine, Connaught Hospital, Ministry of Health and Sanitation, Freetown, Sierra Leone; 2 Comprehensive Care & Support for EVD Survivors, EVD Research, World Health Organization, Freetown, Sierra Leone; 3 34 Military Hospital, Sierra Leone Ministry of Defence, Freetown, Sierra Leone; 4 Viral Special Pathogens Branch, Division of High Consequence Pathogens and Pathology, National Center for Emerging and Zoonotic Infectious Diseases, Centers for Disease Control and Prevention, Atlanta, Georgia, United States of America; 5 Department of Reproductive Health and Research, Family, Women's and Children's Health, World Health Organization, Geneva, Switzerland; 6 National Institute for Viral Disease Control and Prevention, Chinese Center for Disease Control and Prevention, Beijing, People's Republic of China; 7 Sierra Leone-China Friendship Biological Safety Laboratory, Chinese Center for Disease Control and Prevention, Freetown, Sierra Leone; 8 Strategic information, Joint United Nations Programme on HIV/AIDS, Freetown, Sierra Leone; 9 Social & Behavioral Research and Evaluation Branch, Division of Sexually Transmitted Disease Prevention, National Center for HIV/AIDS, Viral Hepatitis, STD, & TB Prevention, Centers for Disease Control and Prevention, Atlanta, Georgia, United States of America; 10 Office of Global Activities, Division of Sexually Transmitted Disease Prevention, National Center for HIV/AIDS, Viral Hepatitis, STD, & TB Prevention, Centers for Disease Control and Prevention, Atlanta, Georgia, United States of America; 11 HIV Care and Treatment Branch, Division of Global HIV/AIDS and Tuberculosis, Center for Global Health, Centers for Disease Control and Prevention, Atlanta, Georgia, United States of America; 12 Pandemic and Epidemic Disease Department, Outbreaks and Health Emergencies, World Health Organization, Geneva, Switzerland; 13 Division of Global Health Protection, Center for Global Health, Centers for Disease Control and Prevention, Atlanta, Georgia, United States of America; The University of Kansas, UNITED STATES

## Abstract

**Background:**

The 2013–2016 West African Ebola virus disease epidemic was unprecedented in terms of the number of cases and survivors. Prior to this epidemic there was limited data available on the persistence of Ebola virus in survivors’ body fluids and the potential risk of transmission, including sexual transmission.

**Methodology/Principal findings:**

Given the urgent need to determine the persistence of Ebola virus in survivors’ body fluids, an observational cohort study was designed and implemented during the epidemic response operation in Sierra Leone. This publication describes study implementation methodology and the key lessons learned. Challenges encountered during implementation included unforeseen duration of follow-up, complexity of interpreting and communicating laboratory results to survivors, and the urgency of translating research findings into public health practice. Strong community engagement helped rapidly implement the study during the epidemic. The study was conducted in two phases. The first phase was initiated within five months of initial protocol discussions and assessed persistence of Ebola virus in semen of 100 adult men. The second phase assessed the persistence of virus in multiple body fluids (semen or vaginal fluid, menstrual blood, breast milk, and urine, rectal fluid, sweat, saliva, tears), of 120 men and 120 women.

**Conclusion/Significance:**

Data from this study informed national and global guidelines in real time and demonstrated the need to implement semen testing programs among Ebola virus disease survivors. The lessons learned and study tools developed accelerated the implementation of such programs in Ebola virus disease affected countries, and also informed studies examining persistence of Zika virus. Research is a vital component of the public health response to an epidemic of a poorly characterized disease. Adequate resources should be rapidly made available to answer critical research questions, in order to better inform response efforts.

## Author summary

The 2013–2016 West African Ebola virus disease epidemic was unprecedented in the numbers of cases, deaths and survivors. Prior to this epidemic, limited data were available about the persistence of Ebola virus in body fluids of Ebola virus disease survivors and the related risk of transmission, including sexual transmission through survivors’ semen. As more data were urgently needed, a study was implemented in Sierra Leone during the epidemic response operation. When establishing this study, many factors were unknown and the findings needed to be urgently translated into practice. This publication summarizes the methodology used for study implementation and the key lessons learned. Strong community engagement helped rapidly initiate this study despite the difficult circumstances of an overwhelming epidemic in a country with few experienced researchers. Implementation was in two phases, first investigating the persistence of Ebola virus in semen only (100 adult men), and secondly, assessing its persistence in multiple body fluids (semen or vaginal fluid, menstrual blood, breast milk; and urine, rectal fluid, sweat, saliva, tears) of an additional 120 men and 120 women. Data from this study immediately informed national and global guidelines and demonstrated the need for semen testing programs. The lessons learned and study tools developed, supported rapid program implementation in Liberia and Sierra Leone.

## Introduction

The 2013–2016 West African Ebola virus disease (EVD) epidemic was unprecedented in terms of the number of cases, deaths [1] and socio-economic consequences across the three most affected countries: Guinea, Liberia and Sierra Leone [2, 3]. As of 10 June 2016, 28,616 confirmed, probable and suspected cases of EVD and 11,310 deaths were reported [1], with over 10,000 survivors documented [1].

Prior to this epidemic, data about the persistence of Ebola virus (EBOV) in survivors’ body fluids and its potential risks for transmission [4, 5] was limited. Based on the limited available evidence, both the World Health Organization (WHO) and the United States Centers for Disease Control and Prevention (CDC) interim guidelines on sexual practices for EVD survivors made a key recommendation of abstinence or correct and consistent condom use for three months post-discharge [6]. During the epidemic there was concern about the risk of Ebola virus transmission from survivors’ semen or other body fluids, especially after several clusters of cases occurred where sexual transmission from an EVD survivor appeared to be the most likely mode of infection [7–10]. Applicable international interim guidelines were immediately updated [11], with WHO recommending that survivors without access to semen testing abstain, or practice correct and consistent condom use for *at least* six months post-symptom onset. US-CDC recommended abstinence or safe sexual practices until additional evidence was available to update guidelines. EVD survivors were facing many challenges, including fear of possible virus transmission to their loved ones, short and long term health sequelae, as well as profound stigma from their communities. This stigma resulted in acute socio-economic consequences including loss of employment, loss of housing and rejection by family members [12–15]. These concerns led to a demand and sustained interest from the survivor community and the public health authorities, for better understanding of EBOV persistence after recovery from acute infection.

An observational cohort study rapidly commenced in Sierra Leone to investigate the presence of EBOV in semen and other body fluids of EVD survivors. When developing this study, many challenges were encountered, including the unknown duration of follow-up required, the potential infectiousness of specimens, the complexity of interpreting and communicating laboratory results, the urgency of translating the research findings into public health practice, and the community understanding and accepting such a study. Thus, given the scale and challenges of conducting the study during the West African EVD epidemic, we describe here the study development, implementation, lessons learned, and how we worked to communicate interim findings, which provided critical evidence to prioritize the development of semen testing programs. The results of the study will be published separately.

## Methods

### Ethics statement

The study was approved by the Sierra Leone Ethics and Scientific Review Committee and the WHO Ethical Review Committee (No. RPC736).

### Study partnerships

The Sierra Leone Ministry of Health and Sanitation (MOHS) led the development and implementation of this study, with technical support from other government and international agencies. The WHO and the CDC were identified as study partner organizations to oversee the development of the study protocol. Steering and technical committees were established (S1 Box) and met regularly to guide the scientific objectives and the implementation of the study. The steering committee identified the Ministry of Defence as the implementing partner. As the CDC diagnostic laboratory in Sierra Leone closed before the study concluded, the Chinese Center for Disease Control and Prevention (China-CDC) joined as a laboratory partner.

### Oversight and quality assurance

International Conference on Harmonization of Good Clinical Practice guidelines were followed as much as possible to guarantee safe study implementation, as well as data integrity and validity of the study results. An independent research monitor conducted a monitoring visit within four months of initiating study enrolment and an external quality control auditor visited the China-CDC laboratory in Sierra Leone to confirm the validity of the real-time reverse transcriptase polymerase chain reaction (qRT-PCR) test results. Additionally, an Independent Data Monitoring Committee was established to review the data quality and guide the study analysis (see S1 Box).

### Study design

Although the authors note the importance of acquiring information on the persistence of EBOV in various body fluids within the pediatric population, for ethical reasons this study was limited to adults. The observational cohort study was conducted in two phases. The first phase of the study was rapidly implemented to investigate the persistence of EBOV ribonucleic acid (RNA) in semen using qRT-PCR testing. It was performed in a convenience sample of 100 adult male EVD survivors. With the experience gained during the implementation of this first phase, a second cohort, using convenience sampling from 120 men and 120 women, was subsequently recruited. This was to extend the knowledge on EBOV persistence by assessing its presence in multiple body fluids (semen or vaginal fluid swab, menstrual blood swab, breast milk and urine, rectal fluid swab, sweat swab, saliva (oral swab), tears), using qRT-PCR testing. During the second phase of the study, an attempt was made to recruit EVD survivors living with human immunodeficiency virus (HIV). Venous blood was collected for the second phase of the study to correlate immune responses with persistence of EBOV. Both phases of the study provided an opportunity to assess sequelae among EVD survivors and any correlation with virus persistence. This was done by conducting health status questionnaires at the time of the study visit.

Participants were invited to a baseline visit and then for follow-up visits every two weeks to provide additional body fluid specimens. Follow-up visits continued until body fluids tested qRT-PCR negative for EBOV RNA on two consecutive visits. Upon initial study discharge, participants received durable, forge-proof certificates stating that ‘fragments of Ebola virus were not detected twice in a row’ for the body fluids analyzed.

Whilst the study was ongoing, there was a report of EVD meningitis in a UK-based survivor nine months into convalescence [16]. This raised questions about the frequency of EVD recurrence and its effect or relationship to EBOV persistence in body fluids. Thus, the study team introduced additional follow-up visits at three and six months after initial study discharge in order to investigate if EBOV could later be identified, after it was previously not detected on two consecutive visits. For the three- and six-month follow-up visits, study discharge criteria at each of these time points was one negative qRT-PCR result, rather than the two consecutive negatives required during the initial follow-up of the study. In the event of a positive qRT-PCR result during the additional three- or six-month follow-up visits, visits continued every two weeks thereafter until body fluids tested qRT-PCR negative at two consecutive visits.

### Identification of study sites

The main criteria used to assess potential study sites were (1) the location and access for survivors, including the (2) size of the recent survivor population in the site’s catchment area, (3) the acceptability by survivors to participate in the study at a given location and (4) the proximity to EVD survivor care services. This was in addition to (5) the feasibility of rapid implementation, including the availability of physical space where infection prevention and control (IPC) standards and participant confidentiality could be maintained, and (6) the engagement and technical competence of the local site staff.

Two study sites were selected. The primary site for the first and second phase of the study was 34 Military Hospital (MH34), Freetown, Western Area. This is a 200-bed secondary referral hospital located in an urban area, run by the Ministry of Defence that included an active Ebola Treatment Unit (ETU) during the epidemic. Lungi Government Hospital (LGH), Kaffa Bullom, Port Loko district, was the second site used for the second phase of the study only. This is 77-bed secondary referral hospital run by the MOHS in a semi-rural area.

### Site design and infection prevention and control

**Fig 1:**
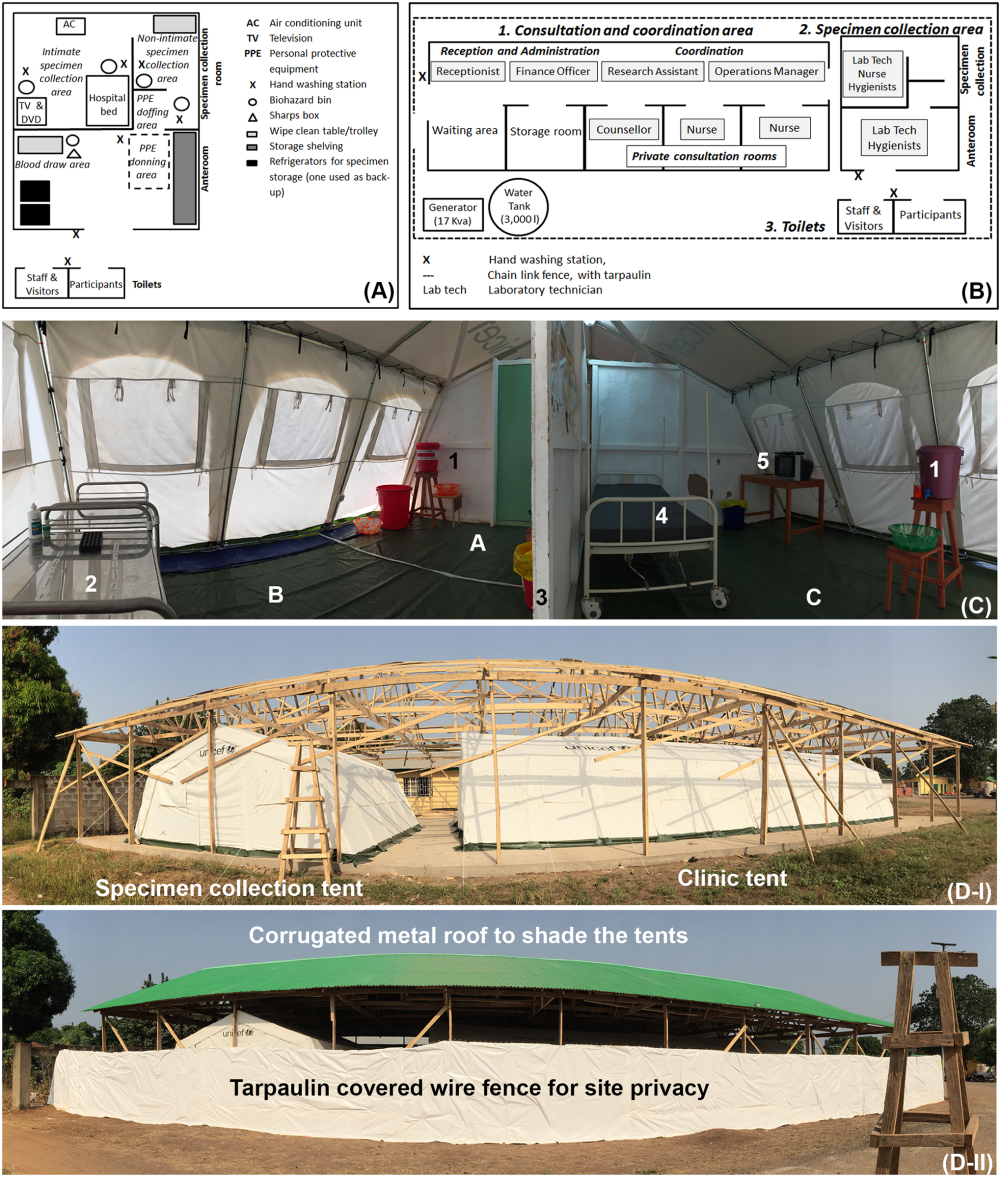
Site design and layout. (A) Schematic of specimen collection tent and toilets. Not to scale. Specimen collection tent: The area was divided into two separate zones; an anteroom (used for storage and a blood draw area), and a private specimen collection room. The second room had a privacy screen for intimate specimen collection. Toilets: The structures (compliant with IPC standards) were clearly identified for either participant use only or staff/visitor use only. At one site portable toilets were utilized and at the other, permanent structures were constructed. (B) Panoramic photograph of the specimen collection room compliant with IPC standards, Lungi Government Hospital site. A). Doffing area (defined with a permanent wooden screen once the site was operational), B). Assisted-collection area (tears, sweat swab, saliva (oral swab), C). Intimate specimen collection area (semen, vaginal/menstrual fluid swabs, rectal fluid swab, breast milk), 1). Hand-washing station, 2). Wipe-clean specimen collection trolley, 3). Privacy screen (which also displayed wipe-clean illustrative posters on specimen collection procedures), 4). Wipe-clean hospital bed, 5). Television set with DVD player, displayed on a wipe-clean table. Tarpaulin flooring used throughout. Photograph by Antoine Coursier. (C) Schematic of site layout and demarcation of work spaces for each staff position. 1). Consultation and coordination area. Reception area: This area was located at the entrance of the study site so the receptionist could greet the participants upon arrival. Waiting area: This area consists of chairs and a television. Community Liaison Officers used this space to informally speak with participants about study questions. Coordination area: Area used for storage of all paper documents (locked filing cabinets), study supervision and data entry. Consultation rooms: These provided audio and visual privacy. To maximize efficiency of participant flow, two rooms were used by nurses to obtain written informed consent and administer questionnaires; one was used for counselling sessions. 2). Specimen collection area. See Fig 1A for details. 3). Toilets. Water tanks supplied the site toilets which were connected to the hospital septic tank at MH34 site; and a purpose built septic tank at LGH site. (D) Photographs documenting the development of the second site at Lungi Government Hospital. (D-I) Separate Rubb Hall tents used for clinic and specimen collection areas. Photograph by Antoine Coursier. (D-II) Measures put in place to maximise privacy and to shield the site from the sun. Photograph by Antoine Coursier.

Due to the lack of adequate existing structures, temporary clinical research sites were constructed. Upon study completion the research sites will be recommissioned for clinical purposes by the respective hospitals. Plastic coated canvas Rubb Hall tents, (often used in humanitarian emergencies), with either wipe-clean tarpaulin or plastic flooring were erected over concrete foundations. At the LGH site, a corrugated metal sheet roof was built to protect the site from the sun and reduce the temperature inside the tents (Fig 1D (ii)). Both sites were designed to facilitate optimal IPC standards and maximize participant privacy (Fig 1A, 1B and 1C). One large tent was partitioned into consultation rooms, office space, and a waiting area, and a smaller tent was used for specimen collection (Fig 1C and 1D (i)). Study sites had separate toilets for staff and participants (the latter being compliant with IPC standards). All body fluids from EVD survivors were assumed to be potentially infectious. The specimen collection tent was divided into an anteroom (for blood drawing, donning of protective equipment, temporary storage of properly packaged specimens) and a specimen collection room (Fig 1A and 1B). The designated specimen collection room was used for all body fluid collections and was treated as a zone requiring personal protective equipment (PPE) for staff that entered the room.

Consultations with IPC experts were conducted in order to facilitate a study site design that would optimize safety and decrease the risk of possible exposure to viable virus. Inspections were performed on several occasions by IPC specialists external to the study. Specimen collection and processing procedures were developed to reflect the IPC requirements for EBOV, which included all items within the specimen collection tent to be (or modified to be) wipe-clean. Relevant staff (laboratory technicians, hygienists, and specimen collection nurses) were trained in IPC practices and all hygienists (who were responsible for decontamination procedures after each participant had completed specimen collection) had relevant and extensive ETU red zone experience prior to joining the study. IPC measures were monitored daily by designated study staff. All standard operating procedures (SOP) including those on IPC procedures, in addition to all study materials (listed in S1 Table) are available on request at reproductivehealth@who.int.

### Staff recruitment and training

The study staff at each site was comprised of around 20 trained personnel (S2 Table), preferably with previous ETU experience. All study staff were trained on site, on each of the specific components of the study (S3 Table), and there was a strong emphasis on sensitivity training and the reduction of stigma and discrimination. Refresher trainings were conducted as required over the course of the study. All national site staff completed International Conference on Harmonization Good Clinical Practice training and laboratory technicians received training on Good Laboratory Practice training and Shipping of Category A (Infectious Substance Affecting Humans) and Category B (Biological Substance) agents.

### Participant recruitment and eligibility

To help minimize the stigma faced by EVD survivors, recruitment processes were carefully planned. The study team led several informational meetings with key stakeholders, including members of the national EVD survivor advocacy group (Sierra Leone Association of Ebola Survivors; SLAES), to introduce the concept, importance and urgency of the study. SLAES supported hiring and training male and female EVD survivors as the study ‘community liaison officers’ (CLOs). The primary function of the CLOs were to engage the survivor community at the group level by conducting sensitization and recruitment meetings, and also at the individual level, by explaining the study to potential participants and facilitating enrollment and retention. CLOs were available on site during study visits to listen to any participants concerns and help answer their questions.

For the first phase of the study (semen only), male participants were recruited through meetings held in collaboration with SLAES and other survivor support groups. For the second phase of the study (multiple body fluids), both men and women were recruited through meetings held in two sessions. The first session addressed both men and women and discussed the study rationale, what to expect during participation, and the collection of non-intimate specimens. The second session was divided by sex, led by a same sex CLO, and discussed collection of intimate or sex-specific specimens (rectal fluid, semen or vaginal fluid, menstrual blood, breast milk). Additional recruitment efforts focused on liaising with active ETUs in order to recruit survivors upon discharge.

EVD survivors aged 18 years or older, who held an ETU discharge certificate and photo identification were eligible for recruitment. Preferentially recruited survivors were those either most recently discharged, or least recently discharged from an ETU (i.e. recent and long term, not mid-term survivors), as well as any pregnant or lactating women. For the targeted recruitment of people living with HIV, the Network for HIV Positives in Sierra Leone was engaged, through collaboration with UNAIDS.

### Participant visits

The content of the different visits are summarized in Table 1.

**Table 1:** Components of the different study visits.

	Baseline visit	Follow-up visits^a^	
		Additional specimen collection	Discharge^b^
Written informed consent^c^ and enrollment	X		
Assignment of a unique study identification number	X		
Standardized questionnaire^c^ Socio-demographics^d^, Ebola virus disease (EVD) health status during and after EVD, sexual history (current/since last study visit)	X X	X	X
Pre- EBOV-test counselling^c^	X	X	
Post EBOV-test counselling^c^ according to test results		X	X
Collection of body fluids	X	X	
Risk-reduction behavioral counselling^c^	X	X	
Optional HIV counselling^c^ and testing	X	X	X
Optional pregnancy testing (when applicable)	X	X	X
Medical referral (when applicable)	X	X	X

^a^ During initial set of visits, and three and six months after initial discharge.

^b^ Participants were discharged when semen/all body fluids tested qRT-PCR negative for EBOV twice consecutively during the initial set of visits, and once during the three and six month follow-up after initial discharge.

^c^ Performed in a language of the participants choice,

^d^ baseline visit only.

During the second phase of the study, staff explained to participants how different body fluid specimens should be collected using illustrative posters and gave instructions on how to follow IPC procedures during collection. Whenever possible, the collection of body fluids followed an order pre-defined in a SOP. The order was based on the likelihood of contaminating another specimen and the intimacy of the specimen being collected. For example to avoid contamination with semen, participants provided their urine specimen first, self-collected in a designated, private, toilet which was compliant with IPC standards. All other body fluids were collected in the specimen collection tent, which was divided into ‘non-intimate’ and ‘intimate’ specimen collection areas using a privacy screen (Fig 1A and 1B). A staff member of the same sex as the participant assisted in the collection of saliva (oral swab), tears, and sweat swab. Then, intimate specimens (rectal fluid swab, vaginal fluid swab, menstrual blood swab, and breast milk) excluding semen, were either self-collected, or collected with assistance as desired. For semen collection, verbal instructions were given about self-collection and all staff left the vicinity during the collection process. The collection area (Fig 1A and 1B) was temperature controlled with the following provided: television and DVD player for viewing pornography if desired by the participant, water-based lubricant and a hospital bed. A radio turned to full volume in the anteroom was used to maintain audio privacy. See Fig 1A–1C for more details on IPC and participant privacy.

All female participants were offered a pregnancy test at each visit. If accepted, testing was conducted by the nurse in the specimen collection room during the body fluid collection stage of the visit, with the results relayed by the counsellor during the same visit. All pregnant participants were referred to antenatal care and all pregnant or lactating participants received nutritional support, including ready-to-use-therapeutic food. An SOP was in place in the event that a qRT-PCR test performed on a breast milk specimen detected the presence of EBOV RNA. In that scenario, the woman would have been immediately instructed to switch from breastfeeding to replacement feeding using ready-to-use infant formula[17], with ongoing counseling and support provided by MOHS Food and Nutrition Directorate staff or a trained study counselor. Ready-to-use infant formula was available onsite to be immediately provided free of charge.

As needed, participants were referred to available clinical services including clinical survivor services for post-recovery complications. Specific referral pathways were designed in collaboration with EVD survivor clinics and specialty medical services, including ophthalmic, mental health and HIV care provided by private and national healthcare pathways. At each visit, the participants received a fixed financial compensation; the amount was determined by the MOHS to cover the average cost of a meal and transport, plus additional money to compensate for loss of earnings due to the time spent at the study clinic.

### Specimen processing and laboratory testing

Once specimen collection from an individual was completed for the visit, specimens were packaged in compliance with international requirements for Category A infectious agents and the room was decontaminated. Following appropriate bio-safety precautions, all specimens were refrigerated within 10 minutes of collection and transported periodically from the study site to the laboratory with cold chain maintained throughout. Specimens were processed within 24–72 hours of collection.

All specimens were tested for EBOV RNA by qRT-PCR in Sierra Leone at either the CDC laboratory (Bo, Bo District) [18–20] or China-CDC laboratory (Jui, Western Area) [21, 22]. At the laboratory, specimens were inventoried, aliquoted into three (if volume was sufficient) and stored in a liquid nitrogen dry shipper, or in a -80°C freezer as available. EBOV isolation was performed on qRT-PCR positive semen specimens from the first phase of the study (semen only), and on positive specimens other than semen from the second phase of the study (multiple body fluids). Unlike qRT-PCR, which was performed in Sierra Leone between 24–72 hours after collection, virus isolation was not available in-country. Therefore one aliquot of each of the specified qRT-PCR positive specimens were packed according to Category A biohazards protocols and transported to CDC in Atlanta, GA, USA, for virus isolation within a biosafety level-4 laboratory [5]. Sequencing of selected specimens from the first phase of the study was also performed in Atlanta, and at China-CDC laboratory (Jui, Western Area, Sierra Leone) for selected specimens from the second phase of the study. The third aliquot of each specimen remained in-country, under the custody of the MOHS.

Blood specimens collected during the second phase of the study, were tested in the China-CDC laboratory to assess both antibodies titers and cellular immune response via immunoglobulin (Ig) G (IgG) and IgM enzyme linked immunosorbent assays (ELISAs) and interferon gamma (IFN-γ) enzyme linked immunosorbent spot (ELISPOT)) assays [23]. Detailed laboratory methods will be published separately.

A data flow system was established between the study sites and the laboratory to reduce manual data entry. A paper and an electronic copy of the specimen list accompanied each specimen transport to the laboratory. Results of qRT-PCR assays were provided electronically within one week and imported into the database to ensure timely and accurate relay of results to participants at their follow-up visits. The results of EBOV isolation were often received months after specimen collection; when available, positive virus isolation results were reported back to participants with additional counselling.

## Challenges, successes, lessons learned and recommendations

### Timeline

Developing and implementing studies usually takes time. It is noteworthy that despite the extremely difficult circumstances faced during the EVD epidemic, the Sierra Leone Ebola Virus Persistence Study was able to commence study enrolment within five months of initial protocol discussions (May 2015), and publish baseline results [24] within 10 months of those discussions (October 2015). The time frame between study enrolment and symptom onset varied widely, with very few participants enrolled within a month of ETU discharge. However, results from this study were key to informing the overall epidemic response.

In the case of diseases for which there is a lack of knowledge, it is essential that research is part of the immediate response to emergency and epidemic situations. Research can be rapidly implemented by having the following key mechanisms in place: (1) funding to address key research questions; (2) ready-made generic protocols available, pre-approved by ethical committees from global institutions such as WHO, so that they can be adapted and submitted to different committees for fast tracked approval; and; (3) flexibility within the research protocol to adapt as new knowledge becomes available. During epidemics, it is important that ethics committees have the capacity to frequently and rapidly expedite clearance of protocol revisions to ensure their timely implementation. This would allow such studies to be implemented, the results obtained, and the response to be informed more rapidly.

### Study design

The criteria of two consecutive negative test results to qualify for study discharge and the two week sampling scheme was a pragmatic decision. This was based on knowledge available at the start of the study on EBOV, and about intermittent shedding in semen of other viruses such as HIV and Hepatitis C virus [25–28]. As noted in the Methods section, the protocol was adapted to accommodate additional three and six month follow-up visits with specimen collection in response to the emergence of novel data [16]. This ability to modify the protocol and be flexible in a rapidly changing environment was an important lesson learned. It highlighted the significance of open communication and good partnerships to ensure timely exchange of new information between response and research. Remaining receptive to new scientific information becoming available and focusing on critical questions related to persistence of EBOV in body fluids allowed the study to provide better informed guidance in tandem to response activities.

### Staffing and community engagement

In general, epidemic response efforts frequently encountered high turnover of staff and vigilant oversight was required to ensure high quality research work. In an emergency setting, it may not always be possible to maintain complete adherence to good management practices, but as a minimum, implementing written SOPs, procedure logs, verbal and written training sessions conducted periodically for all staff, and quality control procedures must be a high priority.

Many of the national staff were either EVD survivors or had ETU experience, and so were sensitive to EVD-related stigma. This was critical in facilitating community engagement and fine-tuning study tools, such as questionnaires and counselling scripts. For staff directly involved with specimen collection, prior ETU experience helped ensure they had the technical expertise and knowledge of IPC standards to conduct safe handling of biohazardous materials. It was essential that CLOs were EVD survivors so that they could relate to the participants. Referral to physicians with specific EVD-related clinical experience helped ensure that medical complications could be addressed efficiently. Of note, the physicians at the primary site (MH34) had treated many of the study participants either when they were admitted into the ETU, or at the EVD survivor clinic. These physicians were widely respected and trusted within the survivor community.

Consistent engagement with, and commitment to survivor communities proved to be critical to the study’s success and was achieved via regular meetings. Study results would be periodically presented to survivor communities and key stakeholders through meetings and/or communication workshops. These activities built relationships and trust between the immediate study staff, greater medical community, and survivor networks. This partnership contributed to achieving full recruitment goals with minimal participants lost to follow-up.

Effective community engagement by staff prior to study implementation was essential to conducting a successful study. For example, focus group discussion with potential female participants prior to the second phase of the study was key to gauging acceptability in this cohort and also aided in tailoring specific study tools. The acceptability by survivors to participate in the study at a particular location was given particularly careful consideration. Prior to finalizing the second site selection, consultation meetings were held with a broad demographic of local adult survivors and key stakeholders (including local chiefs and MOHS district officials), to ascertain if transportation to the existing site in Freetown was favorable to establishing a site in the district. Although many benefits of establishing a site in the district were evident to the local survivor community, concerns were expressed that sightings of small gatherings of survivors attending a local healthcare site may raise suspicion within the community and increase survivor stigma. Similar fears were expressed at establishing a site in the green zone of an existing ETU, in addition to associating ETUs with traumatic experiences and therefore these options were ruled out as potential sites. The Freetown site was a three hour journey away which would have guaranteed anonymity from the local community. However by emphasizing to potential participants the importance the study placed on confidentiality, ranging from the site design to the professional conduct of all staff, concerns were addressed.

### Sensitization, recruitment and participation

With regards to study participation, from sensitization through to follow-up visits, there were several lessons learned and resulting recommendations. For example, during the sensitization and recruitment process for the second phase of the study, staff became aware of non-EVD survivors purchasing forged ETU discharge certificates in order to benefit from EVD survivor resources, including financial compensation for participation in research. Therefore it was recommended that establishing a system to verify EVD survivor status beyond checking ETU discharge certificates, should be considered (e.g. cross checking surveillance databases, laboratory databases and survivor registries or conducting serology testing).

In addition, there were requests from sexually active male survivors younger than 18 years to participate in the study. Therefore with due ethical respect, the authors recommend considering inclusion of minors in a semen testing program. Indeed, the Liberian and Sierra Leonean national semen testing programs were available to males aged 15 years and above.

In this largely Muslim country (77%) [29], the recruitment for the first phase of the study and some of the follow-up period, coincided with the holy month of Ramadan. During this time abstinence from any form of sexual activity was required during daylight hours for Muslim participants. Although recruitment and follow-up slowed significantly during this period, the study remained operational by adapting visit scheduling and successfully navigating cultural sensitivities of this time period. This demonstrated how important it was to engage with local religious leaders and community elders for advice on how to proceed.

Survivors frequently expressed a keen interest in the study because they wanted to know the status of their body fluids. This was to reassure themselves and those with whom they were in close physical contact. The study team had not originally planned to issue certificates to participants upon study completion. However in order to help combat stigma, there was a high demand from participants for certificates which demonstrated test negative status of body fluids. In light of this, certificates of study discharge were issued for which the wording (as noted in the Methods study design section) received very careful consideration from the study team. The wording was an intentionally simplistic factual statement, which could be understood by the community and did not provide any guarantee of “clearance”. Confirmation of twice consecutive test negative status was accompanied by the date of study discharge and the date of certificate issue.

### Messaging and communication

Developing EVD risk reduction counseling messages was challenging due to limited data on the subject [4], the rapid evolution of knowledge as the study progressed, the need to adapt messages to multiple body fluids, and the complexity in explaining the significance of a positive qRT-PCR result [24]. For further details see [30] regarding the development and implementation of the Ebola Virus Persistence Risk Reduction Behavioral Counseling Protocol for this study.

In accordance with the original WHO interim guidance on sexual practices, MOHS recommended that all male and female EVD survivors should abstain from sex or use condoms for three months after ETU discharge [6]. These national guidelines were in effect at the start of the study, and most survivors were not aware that WHO guidance had been updated in May 2015, when the peak of the epidemic had passed in the three most widely effected countries [31]. This needed to be take into account in sensitization efforts and during recruitment. As knowledge on virus persistence was scarce [4, 5], the baseline qRT-PCR results from the first phase of the study (semen only), demonstrating qRT-PCR positive results in semen at least nine months post-symptom onset, were of immediate public health interest and were therefore disseminated rapidly [24]. However the detection of viral RNA by qRT-PCR did not necessarily indicate that infectious virus was present [32, 33] and there is limited data to conclude the correlation between qRT-PCR cycle-threshold (Ct) value and positive virus isolation in semen [32, 34]. Within the context of a public health emergency and the knowledge gaps related to virus persistence, the evolving messaging had to be clearly presented to the various relevant audiences (staff, the survivor community, the scientific community, and at the policy-making level), in a way which avoided further stigmatization of EVD survivors and their sexual partners. An important consideration was that although the interim data generated by the study was groundbreaking, data collection was still ongoing and the number of participants on which the data was based, was limited. The evolving data, combined with epidemiological investigations into potential sexual transmission cases, led to WHO interim guideline updates during the epidemic (January 2016). This included the recommendation that in survivors without access to semen testing, abstinence or correct and consistent condom use should be adopted for at least 12 months after the onset of symptoms [35]. The study team was committed to communicating the sensitive data in real-time, which sometimes proved challenging, when it appeared misaligned with international policy.

The authors recommend that future semen testing initiatives engage with local EVD survivor advocacy groups, communications and social mobilization experts, and key national stakeholders. Maintaining the involvement of these groups throughout the study via regular meetings is crucial to communicating study findings and policy updates, and to ensure consistent messaging is disseminated efficiently. It is critical that communication of study findings and the process of transforming these findings into policy be led by the Ministry of Health and local government agencies.

### Informing other research or programs

Participants commonly viewed participation in the study as receiving an important service. This, along with the initial findings of the study demonstrating a high proportion of qRT-PCR positive test results in semen [24], and the occurrence of new clusters of EVD cases within Sierra Leone potentially linked to transmission from sexual contact with male EVD survivors, were key elements that prompted the government to establish and accelerate the implementation of a semen testing program. WHO, CDC and other partners also engaged with the Ministries of Health in Liberia and Guinea to establish and implement National Semen Testing Programs with preventive behavioral counselling. The research study was also instrumental in accelerating the implementation of these programs by disseminating study SOPs and data capture tools, hosting study site visits, and facilitating training of program staff. Likewise, this was done for other EBOV research studies [e.g. the ‘Ebola Vaccine Ring Vaccination Trial in Guinea’ (Trial number PACTR201503001057193)] and studies on persistence of Zika virus (‘Zika Virus Persistence in Body Fluids of Patients with Zika Virus Infection in Puerto Rico (ZiPer Study)’].

### Specimen collection

The study sites were designed to be safe for specimen collection, with high standards of IPC maintained and no high risk occupational exposures reported. There were various challenges with collecting specimens for particular body fluids. Occasionally participants had difficulty providing tear or sweat specimens at a given visit, but more widely, there were challenges with providing semen or blood specimens.

With regards to semen collection, despite requests from some participants for home or assisted sampling (i.e. in presence of a female companion), it was a study policy to self-collect the specimen on-site. This was in order to (1) decrease the risk of exposing others to any viable EBOV, (2) decrease specimen contamination, (3) protect the viability of any EBOV by maintaining the cold chain, (4) and ensure the specimen was provided by that particular individual. Successful collection of semen specimens was enabled through the careful site design and professional conduct of the staff throughout the site. Some participants had difficulty producing a semen specimen on site for which additional counselling and/or referrals to additional medical care were offered.

A number of study participants were reluctant to have blood collected and shared feelings of mistrust concerning how the blood would be used. The following messages were communicated, which helped to ease fears and facilitate collection: (1) blood would not be sold; (2) all specimens would remain under the ownership of MOHS; (3) only a 4mL vial would be drawn, much less than was collected for other research studies or trials conducted in-country; (4) blood would not be tested to see if participants had EVD again, but used to examine immune response since they were sick with EVD.

### Laboratory

During the course of study implementation, qRT-PCR testing switched from one laboratory group to another. The CDC laboratory performed qRT-PCR testing of all specimens collected throughout the first five months of study operations, at a temporary diagnostic field laboratory located within the grounds of an ETU in Bo District [18]. Prior to this epidemic there had been limited testing of semen for the presence of EBOV. However CDC was able to perform the initial work due to the availability of trained staff who had evaluated their qRT-PCR assay prior to this study to detect EBOV RNA in semen [34]. However, when the epidemic waned, the ETU was decommissioned and this laboratory closed in October 2015. The China-CDC laboratory, located in Western District, was a permanent, purpose-built category 3 laboratory that was constructed during the response effort. China-CDC joined the study team as they had the capacity and sustained presence to maintain qRT-PCR testing for the duration of study operations. A comparison between the qRT-PCR assays performed at these two laboratories was conducted leading up to the laboratory closure in Bo, and testing of study specimens transferred to the China-CDC lab. Details of qRT-PCR assay validation and laboratory data arising from this study will be published separately.

### Conclusion

Rapidly implementing a high quality study during an EVD epidemic in a low-income country, within the context of an overwhelmed and weak health system and few experienced researchers was challenging. The process required balancing limited resources for research with competing response priorities in an ever-changing environment. However, such studies are feasible when led by Government ministries, national staff and community leaders, assisted by solid technical support, and driven by involvement of EVD survivors. This study was a collaborative, multi-organizational effort with the primary goal of supporting the national outbreak response.

Results from this research were crucial for rapidly informing the ongoing response, updating public health recommendations, and potentially preventing new cases. This study demonstrated that the time period between research and program implementation can be substantially reduced during a public health emergency. The data from this study informed national and global recommendations on risk reduction for EVD survivors and their sexual partners during the epidemic and demonstrated the need to implement semen testing services as part of an overall EVD survivor care program. Such semen testing programmes were implemented rapidly in both Sierra Leone and Liberia adapting the methods, mechanisms, and tools developed for the study.

Standardized approaches to studying key issues such as virus persistence during similar epidemics could reduce the time required to have ethical committee approved protocols. As part of the epidemic response planning and resources should be made available and rapidly allocated to address priority research questions. For example, expanding our understanding of virus persistence in the earliest convalescent period after symptom onset and including groups such as pregnant and lactating women, will allow a more accurate assessment of the residual risk of transmission. Commencing research early in an EVD epidemic, as part of the response, will facilitate a better understanding of sexual transmission as well as mother-to-child transmission and will better inform response efforts.

## Supporting information

S1 Box Sierra Leone Ebola Virus Persistence Study—Composition of oversight committees. ETU: Ebola Treatment Unit; EVD: Ebola Virus Disease; HIV: Human immunodeficiency Virus, LGH: Lungi Government Hospital, MH34: 34 Military Hospital, MOHS: Ministry of Health and Sanitation, STIs: Sexually transmitted Infections, *: Also a member of the IDMC secretariat.
(DOCX)

S1 Table The Sierra Leone Ebola Virus Persistence Study, manual of operations, table of contents. For more information, please contact: reproductivehealth@who.int.
(DOCX)

S2 Table Summary of national staff site positions. *One designated driver, in a designated IPC compliant vehicle transported specimens from the study sites to the laboratory. A second driver and vehicle was used to transport site staff and goods to and from the study sites.
(DOCX)

S3 Table Summary of site staff training (34 Military Hospital site). ETU; Ebola treatment unit, EVD; Ebola virus disease, HIV; human immunodeficiency virus, ICF; informed consent form, ICH-GCP; International Conference on Harmonisation-Good Clinical Practice, IPC; Infection prevention control, MOHS; Ministry of Health and Sanitation, PPE; Personal protective equipment, Q&A; Question and Answers, SOPs; Standard operating procedures, UK; United Kingdom.
(XLSX)

S1 Checklist STROBE checklist. (DOC)
