# Supplementary material for: Implementation of a study to examine the persistence of Ebola virus in the body fluids of Ebola virus disease survivors in Sierra Leone: Methodology and lessons learned
Source: PLoS Negl Trop Dis. 2017 Sep 11;11(9):e0005723. doi: 10.1371/journal.pntd.0005723 (PMC5593174; doi:10.1371/journal.pntd.0005723)
Supplement: S1 Box — ETU: Ebola Treatment Unit; EVD: Ebola Virus Disease; HIV: Human immunodeficiency Virus, LGH: Lungi Government Hospital, MH34: 34 Military Hospital, MOHS: Ministry of Health and Sanitation, STIs: Sexually transmitted Infections, *: Also a member of the IDMC secretariat. (DOCX) [file pntd.0005723.s001.docx]

**A. STEERING COMMITTEE** (all members based in Sierra Leone, at least during study implementation)

**Ministry of Health and Sanitation**

- Principal Investigator, Gibrilla F Deen*, (Senior Clinician, Director of Clinical Studies/MOHS Head of Training)
- Study Site Supervisor (LGH), Faustine James*, (Clinician, Hospital Medical Superintendent)
- Study Site Coordinator (LGH), Francis Yamba*, (Clinician)
- Advisor, Amara Jambai, (Deputy Chief Medical Officer, National Lead for Data)
- Advisor, Alie Wurie, (Clinician, National Lead for Survivor Care)
- Advisor, Kwame O’Neil, (Program Manager of Comprehensive Program for EVD Survivors)
- Advisor,* James Bangura, (Western District Lead, EVD Epidemiology)

**Ministry of Defence**

- Study Supervisor (MH34), Foday Sahr*, (Senior Clinician, Commanding Officer, Joint Medical Unit)
- Study Site Coordinator (MH34), Foday R Sesay*, (Clinician, ETU experienced, pioneer of survivor care)
- Assistant Study Site Coordinator (MH34), Thomas A Massaquoi*, (Clinician, ETU experienced, pioneer of survivor care)

**Ministry of Social Welfare, Gender and Children Affairs**

- Advisor, Tina Davies, (National Lead, Child Protection, Gender & Psychosocial for EVD)

**World Health Organization**

- Advisor, Zabulon Yoti, (EVD Specialist, Deputy Country Lead)
- Advisor, Margaret Lamunu*, (EVD Specialist, Technical lead)
- Advisor, Mauricio Calderon, (EVD Survivor Care, Team Lead)
- Advisor, Faiqa Ebrahim, (EVD Survivor Care)

**United States of America Centers for Disease Control and Prevention**

- Advisor, Oliver Morgan*, (Sierra Leone Ebola Response Lead)
- Advisor, Charles Alpren, (Team lead, Survivor Care)
- Advisor, Sara Hersey, (Sierra Leone Country Office Director)

**Joint United Nations Programme on HIV/AIDS**

- Advisor, Patricia Ongpin, (Strategic Information Advisor)

**B. TECHNICAL COMMITTEE**

**World Health Organization**

- Principal Investigator, Nathalie Broutet*, (Epidemiology, STIs), Headquarters
- Lead Technical Advisor, Pierre Formenty* (EVD specialist, Technical Lead for Ebola Response), Headquarters
- Lead Technical Advisor, Anna Thorson*, (Epidemiology, STIs), Headquarters
- Technical Advisor, Kara Durski, (EVD Epidemiology), Headquarters
- Technical Advisor, Dhamari Naidoo, (EVD Laboratory), Sierra Leone and Headquarters
- Study Coordinator (former)/Technical Advisor, Suzanna McDonald*, (Immunology, Clinical Trials, EVD), Sierra Leone
- Study Coordinator, Philippe Gaillard*, (Epidemiology, Clinical Trials), Sierra Leone
- Operations Manager (MH34) (former)/Technical support, Jaclyn Marrinan, (Infectious Disease Specialist), Sierra Leone / Headquarters
- Operations Manager (MH34), Archchun Ariyarajah*, (Epidemiology), Sierra Leone
- Operations Manager (LGH), Antoine Coursier*, (Analyst), Sierra Leone
- Study Support Officer/Temporary Operations Manager, Marylin Carinio, (Public Health), Sierra Leone

**United States of America Centers for Disease Control and Prevention**

- Principal Investigator, Barbara Knust*, (EVD Epidemiology specialist), Headquarters
- Study Operational Support Officer, Elizabeth Ervin, (EVD Epidemiology specialist), Headquarters
- Technical Advisor, Christine Ross, (Epidemiology, HIV), Headquarters
- Technical Advisor, Kyle Bernstein*, (Epidemiology, STIs), Headquarters
- Technical Advisor, Neetu Abad*, (Behavioral science, STIs), Headquarters
- Technical Advisor, Ute Stroher*, (EVD Laboratory specialist), Headquarters
- Technical Advisor, John Klena*, (EVD Laboratory specialist), Headquarters

**Chinese Center for Disease Control and Prevention** (all based in Sierra Leone during study implementation)

- Principal Investigator, Lab Lead (former), Wenbo Xu*, (EVD diagnostics)
- Deputy Lab Lead (former), Hongtu Liu*, (EVD diagnostics)
- Lab Lead (former), William Jun Liu*, (EVD diagnostics)
- Deputy Lab Lead (former), Dapeng Sun (EVD diagnostics)
- Lab Lead, Yong Zhang*, (EVD diagnostics)

**C. INDEPENDENT DATA MONITORING COMMITTEE**

**Board members**

- Clinical EVD Specialist - World Health Organization, Geneva, Switzerland (Ian Crozier)
- Anthropologist - Université Cheikh Anta, Dakar, Senegal (Sylvain Faye)
- Epidemiologist - Karolinska Institute, Stockholm, Sweden (Johan Giesecke)
- Statistician - Harvard T.H. Chan School of Public Health, Boston, United-States of America (Michael Hughes)
- Virologist; EVD Specialist - Public Health Agency of Canada, Winnipeg, Canada (Gary Kobinger)
- Epidemiologist - Centre Muraz, Bobo-Dioulasso, Burkina Faso (Nicolas Meda)
- Virologist - National Institute for Communicable Diseases, Johannesburg, South Africa (Janusz Paweska)
- Statistician - Harvard T.H. Chan School of Public Health, Boston, United-States of America (Donna Spiegelman)
- Behavioral Scientist and HIV Specialist - Muhimbili University of Health and Allied Sciences , Dar es Salaam, Tanzania (Edith Tarimo)

**Secretariat - World Health Organization**

- Statistician (Ndema Habib)
- Executive Director, Outbreaks and Health Emergencies, Director-General's Special Representative for the Ebola Response (Bruce Aylward)
- Assistant Director General, Health Systems and Innovation, Lead for Ebola Research and Development (Marie-Paule Kieny)
- Assistant Director General, Family, Women's and Children's Health (Flavia Bustreo)
- Acting Special Representative and Head of the United Nations Mission for Ebola Emergency Response (UNMEER) (Peter Graaff)
- Headquarters Team Lead, Ebola Epidemiology and Information Management (Chris Dye)
- Director, Department of Reproductive Health and Research, Headquarters (Ian Askew)
- Coordinator for the Human Reproduction Team, Headquarters (James N. Kiarie)
