# Supplementary material for: Implementation of a study to examine the persistence of Ebola virus in the body fluids of Ebola virus disease survivors in Sierra Leone: Methodology and lessons learned
Source: PLoS Negl Trop Dis. 2017 Sep 11;11(9):e0005723. doi: 10.1371/journal.pntd.0005723 (PMC5593174; doi:10.1371/journal.pntd.0005723)
Supplement: S1 Table — For more information, please contact: reproductivehealth@who.int. (DOCX) [file pntd.0005723.s002.docx]

| **A - GENERAL STUDY DOCUMENTS** | **Version date** |
| --- | --- |
| A1-GENERAL STUDY DOCUMENTS - Version Control |  |
| A2-GENERAL STUDY DOCUMENTS - Study Summary |  |
| **B-STUDY COMMITTEES** |  |
| B1-COMMITTEE MEMBERS - Steering Committee |  |
| B2-COMMITTEE MEMBERS - Technical Committee |  |
| B3-COMMITTEE MEMBERS – Independent Data Monitoring Committee |  |
| **C-STUDY STAFF – TERMS OF REFERENCE** |  |
| C01 - Study Site Coordinator |  |
| C02 - Assistant Study Site Coordinator |  |
| C03 - Research Assistant |  |
| C04 - Study Receptionist |  |
| C05 - Study Nurse |  |
| C06 - Study Counselor |  |
| C07 - Study Laboratory Technician |  |
| C08 - Study Hygienist |  |
| C09 - Community Liaison Officer |  |
| C10 - Study Financial Officer |  |
| C11 - Study Driver |  |
| C12-Study-Generator Attendant |  |
| C13 - Study-Night Watchman-House |  |
| C14 - Study-Night Watchman-Site |  |
| C15 - Study-House Keeper |  |
| **D-INFORMED CONSENT** |  |
| D1-INFORMED CONSENT |  |
| **E-STANDARD OPERATING PROCEDURES** |  |
| **E1-SOP CLINIC** |  |
| E1A-SOP CLINIC – Participant Recruitment |  |
| E1A-Appendix 1-CHECKLIST SECOND PHASE OF STUDY-Baseline |  |
| E1A-Appendix 2-CHECKLIST SECOND PHASE OF STUDY-Follow-up |  |
| E1B-SOP CLINIC - Registration, Establishing Eligibility, Managing Appointments |  |
| E1B-Appendix 1-CARD - Follow-Up Appointment |  |
| E1B-Appendix 2-LOG CLINIC - Participant Communication Log (Template) |  |
| E1C-SOP CLINIC - Anthropometric measurements |  |
| E1D-SOP CLINIC - Managing Withdrawals and Loss to Follow-Up |  |
| E1E-SOP CLINIC - Obtaining Informed Consent |  |
| E1F-SOP CLINIC- Administering Questionnaires |  |
| E1G-SOP CLINIC - Serious Adverse Events |  |
| E1G-Appendix 1-LOG CLINIC - Serious Adverse Event |  |
| E1H-SOP CLINIC - Pregnancy Test |  |
| E1H-Appendix 1-Pregnancy Test Sheet |  |
| E1I-SOP CLINIC - Performing HIV Test |  |
| E1I-Appendix 1-HIV Test Sheet for Participant Chart |  |
| E1J-SOP CLINIC - Referring participants to medical services (MH34 E1K-Survivor Clinic, Mental health services, OBS/GYN services) |  |
| E1K-Appendix 1-FORM REFERRAL - 34 Military Hospital |  |
| E1K-Appendix 2-FORM REFERRAL - MH34 Clinic Persistence of Ebola Virus |  |
| E1K-Appendix 3-FORM REFERRAL - Connaught Hospital |  |
| E1L-SOP CLINIC- Disbursement of Incentives |  |
| FORM FINANCE - Authority to Pay |  |
| E1M-SOP CLINIC - Clinic tent temperature monitoring |  |
| **E2-SOP SPECIMENS** |  |
| E2A-SOP Specimens - Specimen Collection Sequence Overview and Specimen labelling |  |
| E2B-SOP SPECIMENS - Blood Collection |  |
| E2B_SOP- SPECIMENS _APPENDIX 1_Poster Blood Collection |  |
| E2C-SOP SPECIMENS - Safe Blood Collection and Disposal of Sharp Objects |  |
| E2D- SPECIMENS SOP - Urine Collection |  |
| E2D_SOP- SPECIMENS_APPENDIX 1_Poster Urine Collection |  |
| E2E-SOP SPECIMENS - Sweat Collection |  |
| E2E_SOP- SPECIMENS_APPENDIX 1_Poster Sweat Collection |  |
| E2F-SOP SPECIMENS – Saliva Collection |  |
| E2F_SOP- SPECIMENS_APPENDIX 1_Poster Saliva Collection |  |
| E2G-SOP SPECIMENS - Tear Collection |  |
| E2G_SOP- SPECIMENS_APPENDIX 1_Poster Tear Collection |  |
| E2H-SOP SPECIMENS - Breast Milk Collection |  |
| E2H_SOP- SPECIMENS_APPENDIX 1_Poster Breast Milk Collection |  |
| E2I-SOP SPECIMENS - Rectal Swab Collection |  |
| E2I_SOP- SPECIMENS_APPENDIX 1_Poster Rectal Swab Collection |  |
| E2J-SOP S SPECIMENS - Semen Collection |  |
| E2K-SOP SPECIMENS - Vaginal Specimen Collection (Vaginal Fluid or Menstrual Blood) |  |
| E2K_SOP- SPECIMENS _APPENDIX 1_Poster Vaginal Specimen Collection (Vaginal Fluid/Menstrual Blood) |  |
| E2L_SOP- SPECIMENS _Pregnancy Test_20160405 |  |
| E2M_SOP- SPECIMENS _Temp Monitoring Cool Box_20160405 |  |
| E2M_SOP- SPECIMENS _APPENDIX 1_Temperature Cool Boxes and Room_2016.03.08 |  |
| E2N_SOP- SPECIMENS _Temp Monitoring_Fridge_20160405 |  |
| E2O_SOP- SPECIMENS _Temp Monitoring_Room_20160405 |  |
| E2P_SOP- SPECIMENS _Specimens packaging_20160405 |  |
| E2P_SOP- SPECIMENS _-APPENDIX 1– Specimens Handling and Specimen Tent Cleaning Log |  |
| E2Q_SOP- SPECIMENS _Study Site to Lab_20160405 |  |
| E2R -SOP_ SPECIMENS COLLECTION - Specimen Collection during 3- and 6-month follow-up |  |
| E2R-Appendix 1-SENDING SPECIMENS- Specimen Line List (Template) |  |
| E2R-Appendix 2-SENDING SPECIMENS- Laboratory Specimen Transport Log (Template) |  |
| **E3-SOP INFECTION PREVENTION & CONTROL** |  |
| E3A-SOP IPC - Cleaning and Disinfection |  |
| E3A-Appendix 1-LOG IPC - CLEANING BATHROOM (TEMPLATE) |  |
| E3A-Appendix 2-LOG IPC - CLEANING STUDY TENT (TEMPLATE) |  |
| E3B-SOP IPC - Medical Waste Management |  |
| E3C-SOP IPC - Donning of PPE |  |
| E3D-SOP IPC - Doffing of PPE |  |
| **E4-SOP CHINA-CDC LAB** |  |
| E4A-SOP CHINA-CDC LAB - Receipt of specimens |  |
| E4B-SOP CHINA-CDC LAB - Preparation of reagents |  |
| E4C-SOP CHINA-CDC LAB - Aliquotting of the specimen |  |
| E4D-SOP CHINA-CDC LAB - Removing the aliquots from containment |  |
| E4E-SOP CHINA-CDC LAB - RNA Extraction by Magmax machine |  |
| E4F-SOP CHINA-CDC LAB - qRT-PCR: Detection Kit for Zaire Ebola Virus RNA (PCR-Fluorescence Probing) |  |
| E4G-SOP CHINA-CDC LAB - Specimen Storage |  |
| E4H-SOP CHINA-CDC LAB - Reporting of qRT-PCR results for data entry |  |
| E4I-SOP CHINA-CDC LAB - Specimen Inventory & Data Management |  |
| **E5-SOP DATA MANAGEMENT** |  |
| E5A-Data Management Strategy |  |
| E5B-SOP DATA - Data checking and verification |  |
| E5C-SOP DATA - Loading database to SharePoint |  |
| E5D-SOP DATA - Transforming and merging Access database to prepare for analysis |  |
| E5E-SOP DATA - Participant files |  |
| E5F-SOP DATA - Enforcing data security |  |
| E5G-SOP DATA - Data entry |  |
| E5H-SOP DATA - Sending data to the lab |  |
| E5I-SOP DATA - Importing lab results |  |
| **F-QUESTIONNAIRE AND TEST FORMS** |  |
| F1-QUESTIONNAIRE - Women - Baseline |  |
| F2-QUESTIONNAIRE - Women – Follow-Up Visit (initial set) |  |
| F3-QUESTIONNAIRE - Women – Follow-Up Visit (3&6 months) |  |
| F4-QUESTIONNAIRE - Men - Baseline |  |
| F5-QUESTIONNAIRE - Men – Follow-Up Visit (initial set) |  |
| F6-QUESTIONNAIRE - Men – Follow-Up Visit (3&6 months) |  |
| F7-QUESTIONNAIRE – Specimen collection – First phase of study |  |
| F8-QUESTIONNAIRE – Specimen collection – Second phase of study |  |
| **G-TALKING POINTS ON INFORMED CONSENT, FAQs, COUNSELLING MATERIAL** |  |
| G1-TALKING POINTS - Informed Consent – Female |  |
| G2-TALKING POINTS – Informed Consent - Male |  |
| G3-FAQS - VIRUS PERSISTENCE STUDY- Community Liaison Officers |  |
| G4-COUNSELLING SCRIPTS |  |
