# Supplementary material for: Implementation of a study to examine the persistence of Ebola virus in the body fluids of Ebola virus disease survivors in Sierra Leone: Methodology and lessons learned
Source: PLoS Negl Trop Dis. 2017 Sep 11;11(9):e0005723. doi: 10.1371/journal.pntd.0005723 (PMC5593174; doi:10.1371/journal.pntd.0005723)
Supplement: S2 Table — *One designated driver, in a designated IPC compliant vehicle transported specimens from the study sites to the laboratory. A second driver and vehicle was used to transport site staff and goods to and from the study sites. (DOCX) [file pntd.0005723.s003.docx]

| **Position** | **Background** | **Number per study site** | **Time commitment** |
| --- | --- | --- | --- |
| Study site supervisor | Medical doctor | 1 | Part time |
| Study site coordinator | Medical doctor | 1 | Part time |
| Finance officer | Experience in finance management | 1 | Part time |
| Receptionist | Nurse or management experience | 1 | Full time |
| Community liaison officer | Ebola virus disease survivor | 2 | Full time |
| Research assistant | Nurse or experience in data management | 1 | Full time |
| Nurse |  | 3 | Full time |
| Counsellor | Training and experience in counselling on HIV infection/reproductive health | 2 | Part time |
| Laboratory technician | Laboratory technician | 2 | Full time |
| Hygienist | Hygienists with prior ETU red zone experience | 2 | Full time |
| Driver* | - | 2 | Full time |
| Night security guard | - | 1 | full time |
| Generator attendant/maintenance | - | 1 | Part time |
