# Supplementary material for: Implementation of a study to examine the persistence of Ebola virus in the body fluids of Ebola virus disease survivors in Sierra Leone: Methodology and lessons learned
Source: PLoS Negl Trop Dis. 2017 Sep 11;11(9):e0005723. doi: 10.1371/journal.pntd.0005723 (PMC5593174; doi:10.1371/journal.pntd.0005723)
Supplement: S1 Checklist — (DOC) [file pntd.0005723.s005.doc]

STROBE Statement—Checklist of items that should be included in reports of ***cohort studies***

|  | Item No | Recommendation |
| --- | --- | --- |
| **Title and abstract** | 1 | (*a*) Indicate the study’s design with a commonly used term in the title or the abstract  **SEE PAGE 3 LINE 49, 54-58**  ‘an observational cohort study was designed and implemented’  ‘The study was conducted in two phases. The first phase was initiated within five months of initial protocol discussions and assessed persistence of Ebola virus in semen of 100 adult men. The second phase assessed the persistence of virus in multiple body fluids (semen or vaginal fluid, menstrual blood, breast milk, and urine, rectal fluid, sweat, saliva, tears), of 120 men and 120 women.’ |
|  |  | (*b*) Provide in the abstract an informative and balanced summary of what was done and what was found  **SEE PAGE 3 LINE 48-65**  ‘Given the urgent need to determine the persistence of Ebola virus in survivors’ body fluids, an observational cohort study was designed and implemented during the epidemic response operation in Sierra Leone. Challenges encountered during implementation included unforeseen duration of follow-up, complexity of interpreting and communicating laboratory results to survivors, and the urgency of translating research findings into public health practice. Strong community engagement helped rapidly implement the study during the epidemic. The study was conducted in two phases. The first phase was initiated within five months of initial protocol discussions and assessed persistence of Ebola virus in semen of 100 adult men. The second phase assessed the persistence of virus in multiple body fluids (semen or vaginal fluid, menstrual blood, breast milk, and urine, rectal fluid, sweat, saliva, tears), of 120 men and 120 women.  Conclusion / Significance Data from this study informed national and global guidelines in real-time and demonstrated the need to implement semen testing programs among Ebola virus disease survivors. The lessons learned and study tools developed accelerated the implementation of such programs in Ebola virus disease affected countries, and also informed studies examining persistence of Zika virus. Research is a vital component of the public health response to an epidemic of a poorly characterized disease. Adequate resources should be allocated to rapidly answer critical research questions, in order to better inform response efforts.**’** |
| Introduction | | |
| Background/rationale | 2 | Explain the scientific background and rationale for the investigation being reported  **SEE PAGE 3 LINE 44-49 // PAGE 4 LINE 67-71 // PAGE 5 LINE 85-90, 93-96**  **‘**The 2013-2016 West African Ebola virus disease epidemic was unprecedented in terms of the number of cases and survivors. Prior to this epidemic there was limited data available on the persistence of Ebola virus in survivors’ body fluids and the potential risk of transmission, including sexual transmission.  Given the urgent need to determine the persistence of Ebola virus in survivors’ body fluid ….’  ‘The 2013-2016 West African Ebola virus disease epidemic was unprecedented in the numbers of cases, deaths and survivors. Prior to this epidemic, limited data were available about the persistence of Ebola virus in body fluids of Ebola virus disease survivors and the related risk of transmission, including sexual transmission through survivors’ semen. As more data was urgently needed, a study was implemented in Sierra Leone during the epidemic response operation.’  ‘The 2013-2016 West African Ebola virus disease (EVD) epidemic was unprecedented in terms of the number of cases, deaths [1] and socio-economic consequences across the three most affected countries: Guinea, Liberia and Sierra Leone [2, 3]. As of 10 June 2016, 28,616 confirmed, probable and suspected cases of EVD and 11,310 deaths were reported [1], with over 10,000 survivors documented [1].  ‘Prior to this epidemic, data about the persistence of Ebola virus (EBOV) in survivors’ body fluids and its potential risks for transmission [4, 5] was limited. During the epidemic there was concern about the risk of Ebola virus transmission from survivors’ semen or other body fluids,’ |
| Objectives | 3 | State specific objectives, including any pre-specified hypotheses  **SEE PAGE 5 LINE 106-107 (STUDY OBJECTIVE),**  ‘An observational cohort study rapidly commenced [in Sierra Leone] to investigate the presence of EBOV in semen and other body fluids of EVD survivors.’  **SEE PAGE 5 LINE 111-114 (PAPER OBJECTIVE)**  ‘Thus, given the scale and challenges of conducting the study during the West African EVD epidemic, we describe the study development, implementation, lessons learned, and how the findings provided critical evidence to support the development of semen testing programs. The results of the study will be published separately.’ |
| Methods | | |
| Study design | 4 | Present key elements of study design early in the paper  **SEE PAGE 3 LINE 49, 54-58 // PAGE 4 LINE 75-78 // PAGE 7-8 LINE 137-163,**  ‘…an observational cohort study was designed and implemented during the epidemic response operation in Sierra Leone.’  ‘The study was conducted in two phases. The first phase was initiated within five months of initial protocol discussions and assessed persistence of Ebola virus in semen of 100 adult men. The second phase assessed the persistence of virus in multiple body fluids (semen or vaginal fluid, menstrual blood, breast milk, and urine, rectal fluid, sweat, saliva, tears), of 120 men and 120 women.’  **PAGE 4 LINE 75-78:** ‘Implementation was in two phases, first investigating the persistence of Ebola virus in semen only (100 adult men), and secondly, assessing its persistence in multiple body fluids (semen or vaginal fluid, menstrual blood, breast milk; and urine, rectal fluid, sweat, saliva, tears) of an additional 120 men and 120 women.’  **PAGE 6-7 LINE 137-163,**  **‘**for ethical reasons this study was limited to adults**’, (line 137)**  **Please see study design section (137-163).** |
| Setting | 5 | Describe the setting, locations, and relevant dates, including periods of recruitment, exposure, follow-up, and data collection  **SEE PAGE 8, LINE 164-176 (‘Identification of study sites’ section) for setting/location**  **PAGE 11-13, LINE 240-262 (‘Participant recruitment and eligibility’ section).** Data on recruitments dates will be given in ‘results papers’.  **LINE 265 (Table 1 Components of the different study visits) (data collection and follow-up)**  **‘**questionnaire on ‘socio-demographics, Ebola virus disease (EVD) health status during and after EVD, sexual history (current/since last study visit).’ |
| Participants | 6 | (*a*) Give the eligibility criteria, and the sources and methods of selection of participants. Describe methods of follow-up  **SEE PAGE 11 LINE 285-262, (eligibility)**  **‘**EVD survivors aged 18 years or older, who held an ETU discharge certificate and photo identification were eligible for recruitment. Preferentially recruited survivors were those either most recently discharged, or least recently discharged from an ETU (i.e. recent and long term, not mid-term survivors), as well as any pregnant or lactating women. For the targeted recruitment of people living with HIV, the Network for HIV Positives in Sierra Leone was engaged, through collaboration with UNAIDS.**’**  **PAGE 13-14, LINE 271 – 303 (follow-up)**  **‘See Table 1 (Components of the different study visits); line 265’**  **LINE 267-8**  ‘Participants were discharged when semen/all body fluids tested qRT-PCR negative for EBOV twice consecutively during the initial set of visits, and once during the three and six month follow-up after initial discharge.’ |
|  |  | (*b*) For matched studies, give matching criteria and number of exposed and unexposed  **NOT APPLICABLE FOR THIS STUDY** |
| Variables | 7 | Clearly define all outcomes, exposures, predictors, potential confounders, and effect modifiers. Give diagnostic criteria, if applicable  **NOT APPLICABLE FOR THIS PAPER** |
| Data sources/ measurement | 8* | For each variable of interest, give sources of data and details of methods of assessment (measurement). Describe comparability of assessment methods if there is more than one group  **PARTIALLY APPLICABLE, SEE PAGE 14-15 LINE 313-326 (**methods of assessment (measurement))  ‘EBOV isolation was performed on qRT-PCR positive semen specimens from the first phase of the study (semen only), and on positive specimens other than semen from the second phase of the study (multiple body fluids). Unlike qRT-PCR, which was performed in Sierra Leone between 24-72 hours after collection, virus isolation was not available in-country. Therefore one aliquot of each of the specified qRT-PCR positive specimens were packed according to Category A biohazards protocols and transported to CDC in Atlanta, GA, USA, for virus isolation within a biosafety level-4 laboratory [5]. Sequencing of selected specimens from the first phase of the study was also performed in Atlanta, and at China-CDC laboratory (Jui, Western Area, Sierra Leone) for selected specimens from the second phase of the study. The third aliquot of each specimen remained in-country, under the custody of the MOHS.  Blood specimens collected during the second phase of the study, were tested in the China-CDC laboratory to assess both antibodies titers and cellular immune response via immunoglobulin (Ig) G (IgG) and IgM enzyme linked immunosorbent assays (ELISAs) and interferon gamma (IFN-γ) enzyme linked immunosorbent spot (ELISPOT)) assays [17]. Detailed laboratory methods will be published separately.’ |
| Bias | 9 | Describe any efforts to address potential sources of bias  **NOT APPLICABLE FOR THIS PAPER, WILL BE DISCUSSED IN SUBSEQUENT ‘RESULTS PAPERS’** |
| Study size | 10 | Explain how the study size was arrived at  **NOT APPLICABLE FOR THIS PAPER, WILL BE DISCUSSED IN SUBSEQUENT ‘RESULTS PAPERS’** |
| Quantitative variables | 11 | Explain how quantitative variables were handled in the analyses. If applicable, describe which groupings were chosen and why  **NOT APPLICABLE FOR THIS PAPER, WILL BE DISCUSSED IN SUBSEQUENT ‘RESULTS PAPERS’** |
| Statistical methods  **NOT APPLICABLE**  **FOR THIS PAPER, WILL BE DISCUSSED IN SUBSEQUENT ‘RESULTS PAPERS’** | 12 | (*a*) Describe all statistical methods, including those used to control for confounding |
|  |  | (*b*) Describe any methods used to examine subgroups and interactions |
|  |  | (*c*) Explain how missing data were addressed |
|  |  | (*d*) If applicable, explain how loss to follow-up was addressed |
|  |  | (*e*) Describe any sensitivity analyses |
| Results | | |
| Participants  **NOT APPLICABLE**  **FOR THIS PAPER, WILL BE DISCUSSED IN SUBSEQUENT ‘RESULTS PAPERS’** | 13* | (a) Report numbers of individuals at each stage of study—eg numbers potentially eligible, examined for eligibility, confirmed eligible, included in the study, completing follow-up, and analysed |
|  |  | (b) Give reasons for non-participation at each stage |
|  |  | (c) Consider use of a flow diagram |
| Descriptive data  **NOT APPLICABLE**  **FOR THIS PAPER, WILL BE DISCUSSED IN SUBSEQUENT ‘RESULTS PAPERS’** | 14* | (a) Give characteristics of study participants (eg demographic, clinical, social) and information on exposures and potential confounders |
|  |  | (b) Indicate number of participants with missing data for each variable of interest |
|  |  | (c) Summarise follow-up time (eg, average and total amount) |
| Outcome data **NA** | 15* | Report numbers of outcome events or summary measures over time |
| Main results  **NOT APPLICABLE**  **FOR THIS PAPER, WILL BE DISCUSSED IN SUBSEQUENT ‘RESULTS PAPERS’** | 16 | (*a*) Give unadjusted estimates and, if applicable, confounder-adjusted estimates and their precision (eg, 95% confidence interval). Make clear which confounders were adjusted for and why they were included |
|  |  | (*b*) Report category boundaries when continuous variables were categorized |
|  |  | (*c*) If relevant, consider translating estimates of relative risk into absolute risk for a meaningful time period |
| Other analyses  **NOT APPLICABLE** | 17 | Report other analyses done—eg analyses of subgroups and interactions, and sensitivity analyses |
| Discussion | | |
| Key results **NA** | 18 | Summarise key results with reference to study objectives |
| Limitations  **NOT APPLICABLE**  **FOR THIS PAPER, WILL BE DISCUSSED IN SUBSEQUENT ‘RESULTS PAPERS’** | 19 | Discuss limitations of the study, taking into account sources of potential bias or imprecision. Discuss both direction and magnitude of any potential bias |
| Interpretation  **NOT APPLICABLE**  **FOR THIS PAPER, WILL BE DISCUSSED IN SUBSEQUENT ‘RESULTS PAPERS’** | 20 | Give a cautious overall interpretation of results considering objectives, limitations, multiplicity of analyses, results from similar studies, and other relevant evidence |
| Generalisability  **NOT APPLICABLE** | 21 | Discuss the generalisability (external validity) of the study results |
| Other information | | |
| Funding | 22 | Give the source of funding and the role of the funders for the present study and, if applicable, for the original study on which the present article is based  **financial disclosure section of submission**  ‘Funding provided by the World Health Organization (PF NB), the United States Centers for Disease Control and Prevention (BK OM STN), the Chinese Center for Disease Control and Prevention (WX HL WJL YZ GW ML), and the Paul G. Allen Family Foundation (http://www.pgafamilyfoundation.org/) (PF NB AET).’  ‘The World Health Organization gratefully acknowledges the financial contribution of the WHO's Ebola Response Programme (PF), The Paul G. Allen Family Foundation (NB PF AET), and the UNDP-UNFPA-UNICEF-WHO-World Bank Special Programme of Research, Development and Research Training in Human Reproduction (HRP) (NB) in support of the Sierra Leone Ebola Virus Persistence Study.’  ‘The funders had no role in study design, data collection and analysis, decision to publish, or preparation of the manuscript. The views expressed in this article are those of the authors and do not necessarily represent the official positions, decisions, policy or views of the Government of Sierra Leone Ministry of Health and Sanitation, Ministry of Defence, the World Health Organization, the United States Centers for Disease Control and Prevention, or the Chinese Center for Disease Control and Prevention.’ |

*Give information separately for exposed and unexposed groups.

**Note:** An Explanation and Elaboration article discusses each checklist item and gives methodological background and published examples of transparent reporting. The STROBE checklist is best used in conjunction with this article (freely available on the Web sites of PLoS Medicine at http://www.plosmedicine.org/, Annals of Internal Medicine at http://www.annals.org/, and Epidemiology at http://www.epidem.com/). Information on the STROBE Initiative is available at http://www.strobe-statement.org.
